# Aging and rejuvenation - a modular epigenome model

**DOI:** 10.18632/aging.202712

**Published:** 2021-02-24

**Authors:** Priscila Chiavellini, Martina Canatelli-Mallat, Marianne Lehmann, Maria D. Gallardo, Claudia B. Herenu, Jose L. Cordeiro, James Clement, Rodolfo G. Goya

**Affiliations:** 1 Institute for Biochemical Research (INIBIOLP) - Histology B and Pathology B, School of Medicine, National University of La Plata, La Plata, Argentina; 2 Institute for Experimental Pharmacology (IFEC), School of Chemical Sciences, National University of Cordoba, Cordoba, Argentina; 3 World Academy of Art and Science (WAAS), Napa, CA 94558, USA; 4 Betterhumans Inc., Gainesville, FL 32609, USA

**Keywords:** aging, DNA methylation, epigenetic clock, rejuvenation, cell reprogramming

## Abstract

The view of aging has evolved in parallel with the advances in biomedical sciences. Long considered as an irreversible process where interventions were only aimed at slowing down its progression, breakthrough discoveries like animal cloning and cell reprogramming have deeply changed our understanding of postnatal development, giving rise to the emerging view that the epigenome is the driver of aging. The idea was significantly strengthened by the converging discovery that DNA methylation (DNAm) at specific CpG sites could be used as a highly accurate biomarker of age defined by an algorithm known as the Horvath clock. It was at this point where epigenetic rejuvenation came into play as a strategy to reveal to what extent biological age can be set back by making the clock tick backwards. Initial evidence suggests that when the clock is forced to tick backwards *in vivo*, it is only able to drag the phenotype to a partially rejuvenated condition. In order to explain the results, a bimodular epigenome is proposed, where module A represents the DNAm clock component and module B the remainder of the epigenome. Epigenetic rejuvenation seems to hold the key to arresting or even reversing organismal aging.

## INTRODUCTION

Conventionally, age is defined as the period that spans from birth to a given point in time. More rigorously, age may be defined as the time elapsed from conception of a zygote to a given time point. A more relevant concept than chronological age is biological age, which refers to the functional and structural status of an organism at a given age. A number of markers of biological age have been used over the years but none of them achieved sufficient accuracy for practical purposes [1–4]. The situation changed with the relatively recent discovery that the level of age-related methylation of a set of cytosine-guanine dinucleotides (CpG) located at specific positions on DNA throughout the genome, constitutes a highly reliable biomarker of aging defined by a mathematical algorithm, the multi-tissue age predictor also known as the epigenetic clock, devised by Stephen Horvath in 2013 [5]. Other epigenetic clocks have been devised [6–9] but since most are based on DNA methylation (DNAm) profiles, what will be discussed here for Horvath’s clock will apply to all of them. The features of the epigenetic clock have been extensively discussed in the literature [5, 10, 11] and will not be reviewed here. We will discuss different views of aging considered as an epigenetic mechanism, the role that the epigenetic clock may play in the process and rejuvenation as an interventive approach able to set back epigenetic age.

### Homeorrhesis, developmental program and aging

From a physiological viewpoint, aging is characterized by a progressive decline in homeostatic potential with time [12] as well as by a drift in the set point of homeostasis, a process known as homeorrhesis. The term homeorrhesis, or moving homeostasis, was first used by Waddington in 1957 [13]. It refers to the fact that the set point of homeostasis changes during the different phases of life, as do other physiological parameters. What we usually call normal physiology is that of the young, optimally functioning adult, human or animal. From a developmental point of view, however, there is a sequence of normal physiologies, beginning in the egg and ending in the senescent individual. The homeostasis that gives us our frame of reference to define "normal" function is in fact homeostasis about a developmentally changing point.

Although conventionally, the different stages of life are divided into development, adulthood, and aging, from a biological standpoint, all stages of life constitute a continuum and epigenetic age moves along that continuum.

The nature and timing of the sequence of events that begins after fertilization and ends when sexual maturity is attained (developmental phase) must be genetically programmed and epigenetically driven. This program must be highly complex and must involve a great degree of coordination among different groups of cells, especially during the embryonic stage of the developing organism [14].

There are two possibilities after sexual maturity has been reached: (a) the aging process is also programmed; (b) the developmental program runs out shortly after sexual maturation occurs. In the latter scenario, the length of the reproductive period would be determined by the robustness of the homeostatic network of the adult organism. Senescence would appear when the network begins to run over tolerance [15]. The idea that aging is a programmed process has been previously proposed based on experimental data mainly from yeasts (Saccharomyces cerevisiae), worms (Caenorhabditis elegans) and mice [16]. The hypothesis that aging is a programmed process under the control of an epigenetic clock has also been proposed [17]. Another model of programmed aging proposes that organismal aging is the result of the combined action of many clocks, like DNA damage, telomere attrition, mitochondrial production of reactive oxygen and nitrogen species (ROS and RNS) at the cellular/molecular level, as well as higher level clocks located in the central nervous system [18].

A strong source of evidence supporting programmed aging as a viable hypothesis comes from plants. In effect, plants are the only organisms where programmed cell death has a fairly well-established role during senescence (the word aging is seldom used for plants). Furthermore, senescence itself is a programmed event in some plants [19–22]. There are, however, views favoring alternative (b), namely, that aging is not a programmed process but a continuation of developmental growth, driven by genetic pathways such as mTOR [23]. It has also been proposed that biological aging may be a consequence of both developmental and maintenance programs, the molecular footprints of which give rise to epigenetic clocks [10].

### The epigenetic clock and aging

In humans, the epigenetic age calculated by the clock algorithm shows a correlation of 0.96 to chronological age and an error margin of 3.6 years, an unprecedented accuracy for a biomarker of age [5, 24]. Interestingly, the epigenetic clock predicts biological age with comparable high accuracy when applied to DNA taken from whole blood, peripheral blood mononuclear cells, occipital cortex, buccal epithelium, colon, adipose, liver, lung, saliva, and uterine cervix [5, 24]. The rate of change in DNA methylation at age-dependent CpGs represents the ticking rate of the epigenetic clock. The rate is very high in humans from birth to one year of age, from 1 to 20 years of age it progressively decelerates and from age 20 onwards it changes to a much slower rate [5, 10, 24]. It can also be said that the ticking rate of the epigenetic clock represents the changing rate of DNA methylation heterogeneity among cells in tissues [24].

There is compelling evidence that the ticking rate of the clock is significantly correlated with the rate of biological aging in health and disease. For instance, it is known that the rate of epigenetic aging is slower in supercentenarians and their descendants than in non-centenarians [25]. Under pathological circumstances the epigenetic age displayed by the clock represents biological rather than chronological age. In humans, there are a growing number of pathologies associated with accelerated epigenetic aging, where evidence reveals a consistent and highly significant correlation between the rate of epigenetic and biological aging (Table 1). Such a consistent correlation between the rate of epigenetic and organismal aging suggests, although does not prove, that the DNAm clock may be the driver of organismal aging. It should be also considered that the epigenetic pacemaker of aging may be a modular mechanism of which the epigenetic clock is a component (see below).

**Table 1:** Conditions that affect the rate of epigenetic aging in humans.

**Condition**	**Aging rate**	**References**
Down syndrome	Fast	[56]
Vasomotor symptoms	Fast	[57]
Werner syndrome	Fast	[58]
Bipolar disorder	Fast	[59]
Huntington's disease	Fast	[60]
Obesity	Fast	[61, 62]
Menopause	Fast	[63]
Parkinson	Fast	[64]
Cancer	Fast	[65–67]
Centenarian or Supercentenarian	Slow	[24]
Allergy and asthma	Fast	[68]
HIV	Fast	[69–71]
Alzheimer	Fast	[72]
Alcohol use disorder	Fast	[73]
Osteoarthritis	Fast	[74]

Unfortunately, the possibility of moving from the realm of speculation into the field of experimentation seems elusive at present. Any experimental manipulation that either accelerates or slows down the rate of biological aging in an animal model is likely to act in the same sense on the rate of epigenetic aging. However, there would be no way to determine whether the primary effect of the manipulation was on the epigenome or on the downstream components of the DNA machinery.

The above considerations apply to an epigenetic clock that ticks forward, that is in an organism that ages. Since we now have molecular tools, like the Yamanaka factors, that allow us to make the clock tick backwards, the time is ripe for opening a new dimension in gerontology, moving from aging research to epigenetic rejuvenation research. This is the main topic of the next sections.

### Rejuvenation by cell reprogramming

Embryonic stem (ES) cells have the lowest possible chronological and epigenetic age that an organism may have. In biological terms, ES cells are fascinating as they may remain indefinitely young, that is, in a kind of suspended animation even if they keep proliferating. In ES cells, the epigenetic clock does not tick [5] nor does the circadian clock oscillate [26]. Only when ES cells differentiate, both clocks become active and cells begin to age. The first clues that somatic cells can be rejuvenated back to ES-like cells came from the development of animal cloning in the early 60s [27] and more recently, of cell reprogramming [28]. These seminal achievements paved the way for the subsequent implementation of cell rejuvenation [29–31].

There is clear evidence that cell reprogramming rejuvenates cells. Thus, it has been demonstrated that when somatic cells are reprogrammed to induced pluripotent stem (iPS) cells, which are embryonic-like cells, their epigenetic clock stops ticking, and circadian clocks stop oscillating [5, 26]. Furthermore, epigenetic age in human- and mouse-derived iPS cells is set back to zero or near zero, which is consistent with their blastocyst-like characteristics [5, 32].

**Figure 1:**
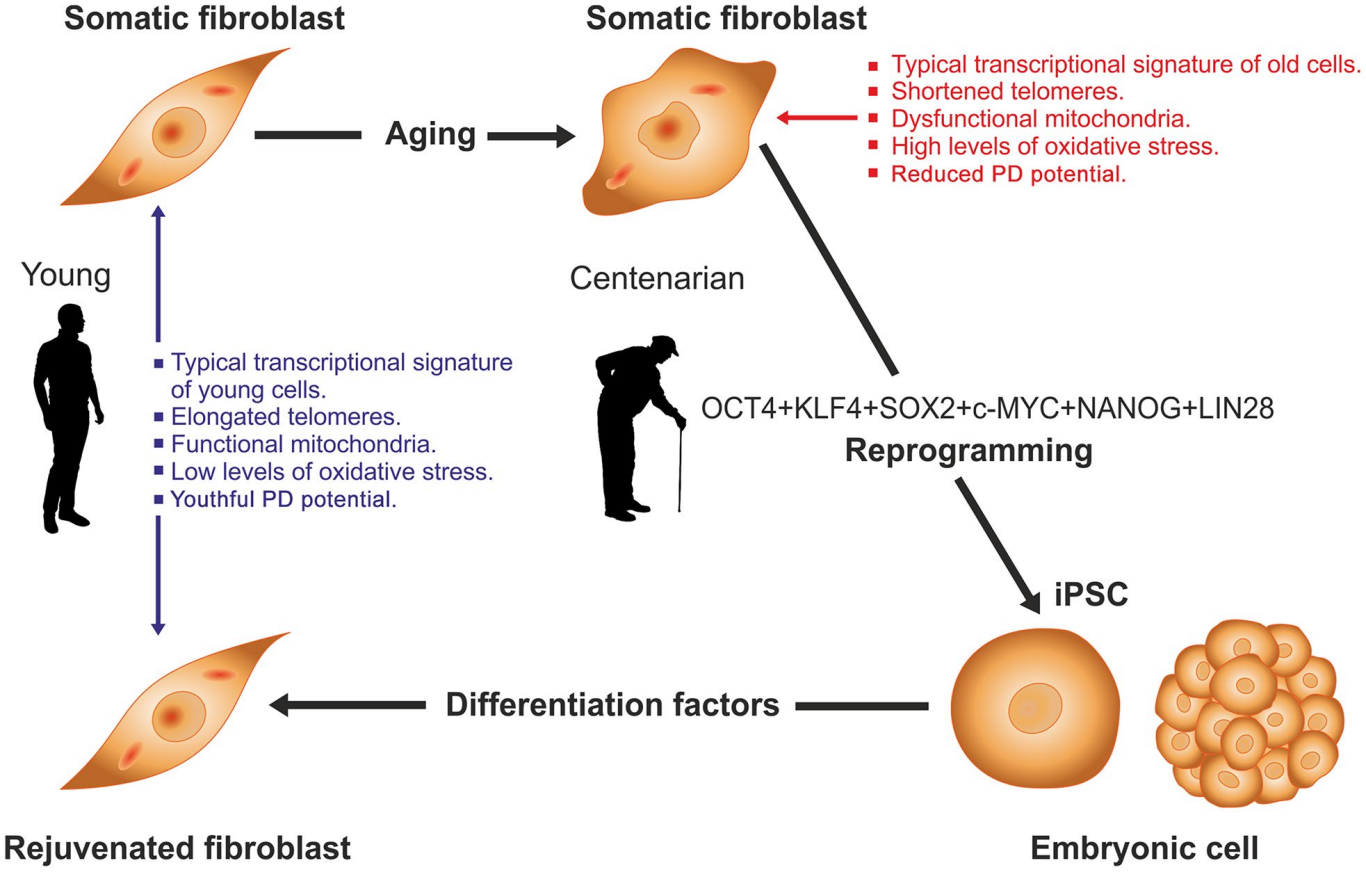
**Rejuvenation by cell reprogramming of fibroblasts from healthy centenarian individuals.** In culture, fibroblasts from old individuals display a typical transcriptional signature, different of that from young counterparts as well as shortened telomeres, reduced population doubling (PD) potential, dysfunctional mitochondria and higher levels of oxidative stress. When cells were reprogrammed to iPS with a 6-factor cocktail the above alterations were fully reversed. Then iPS cells were differentiated back to fibroblasts by culture in the presence of an appropriate set of differentiation factors. In the resulting cells, all of the above variables had levels typical of fibroblasts taken from young individuals. See [34] for further details.

In some instances, reprogramming cells from old individuals requires specific approaches. Thus, skin fibroblasts from old (18 months) mice were rejuvenated into ES cells by somatic cell nuclear transfer (SCNT) followed by collection of the inner cell mass from the resulting embryonic blastulas [33]. If the blastocysts were differentiated back into fibroblasts the newly generated cells would be rejuvenated. Cell rejuvenation has been also achieved in skin fibroblasts from healthy centenarians [34]. When placed in culture, skin fibroblasts from centenarians display a number of morphologic, molecular and functional deficits consequential to the highly advanced age of the donor. In 2011 a French group led by JM Lemaitre was able to reprogram skin fibroblasts from healthy centenarians using a cocktail of six reprogramming genes, Oct4, Sox2, Klf4, c-Myc, Nanog and Lin28 [34]. This 6-factor combination reprogrammed fibroblasts from very old donors into typical iPS cells. The iPS cells so generated were incubated with a differentiation cocktail that induced them to differentiate back into fibroblasts whose transcriptional profile, mitochondrial metabolism, oxidative stress levels, telomere length and population doubling potential were indistinguishable from those of skin fibroblasts from young counterparts (Figure 1). After taking the data together, the authors concluded that the centenarians’ fibroblasts had been rejuvenated. The implications of this and other reports [35–39] are far-reaching and place the view of aging under a new perspective, which in such a novel field, leads to avenues that at present are of necessity hypothetical, essentially new ideas to test. For the sake of clarity, we resort to an analogy presented by Alex Comfort to illustrate the determination of lifespan, which imagines an interplanetary probe whose mission is to fly past Mars [40]. The probe carries an onboard computer that acts as a homeostatic system monitoring the correct functioning of the different components of the probe. In such device the lifespan of each component has been programmed to last until the end of the mission allowing for some tolerance to compensate for any unforeseen extensions of the mission. During the flight, the computer will correct any malfunction consequential to wear and tear of individual components. Upon arrival to Mars (analogous to successful reproduction in biological systems) the probe will continue functioning for some time but its different components will be expected to become progressively damaged by environmental factors and endogenous wear and tear (cumulative damage). The computer software will also be expected to undergo progressive deterioration (loss of information) and at a certain point fuel will be exhausted. The probe would be approaching the end of its functional life. Let’s assume that the probe is somehow refueled and mission control on earth sends to it wireless computer commands to reignite engines and return to earth. If the cumulative damage of computer hardware and loss of information in the software have gone beyond a critical point, the probe will not be able to effectively respond to mission control telecommands. But if the probe responds appropriately to all commands and successfully returns to earth, the engineers that built the device will be compelled to conclude that the integrity of the critical components and the information on the computer were fully preserved. Cell rejuvenation studies like the two summarized above, suggest that even at advanced ages the epigenome remains responsive to command signals like the OSKM genes which is compatible with the hypothesis that aging, even at advanced stages, is not associated with critical deterioration of the epigenetic mechanisms that control cell function. Recently, an alternative hypothesis has been drawn from studies in transgenic mice known as ICE mice (for inducible changes to the epigenome). In these animals several aging features can be attained by inducing non-mutagenic double breaks in DNA which disrupt epigenetic information. The results led to the view that aging is not due to DNA mutations but to progressive epigenome disorganization and consequential loss of epigenetic information [41, 42].

The possibility is also considered that a backup copy of epigenetic information could be stored somewhere within cells. In order to explain the ability of cells from aged individuals to be reprogrammed into iPS cells in the framework of this alternative hypothesis [43], one must assume that cells can retrieve this putative backup information in response to reprogramming factors. An implication of the epigenetic backup hypothesis is that in mammals, a complex molecular mechanism evolved to keep a backup copy of epigenetic information in somatic cells. It is difficult to imagine what evolutionary purpose such storage mechanism may serve, as rejuvenation does not occur naturally in mammals. In this context, it seems reasonable to apply the philosophical principle known as the Occam’s razor or simplicity principle, which states that when there are two or more alternative hypotheses to explain an occurrence, the one that requires the smallest number of assumptions is likely to be correct [44]. In this case, the simplest hypothesis is that aging, even at advanced stages, is not associated with critical deterioration of the epigenome.

### Rejuvenation by cyclic partial cell reprogramming

**Figure 2:**
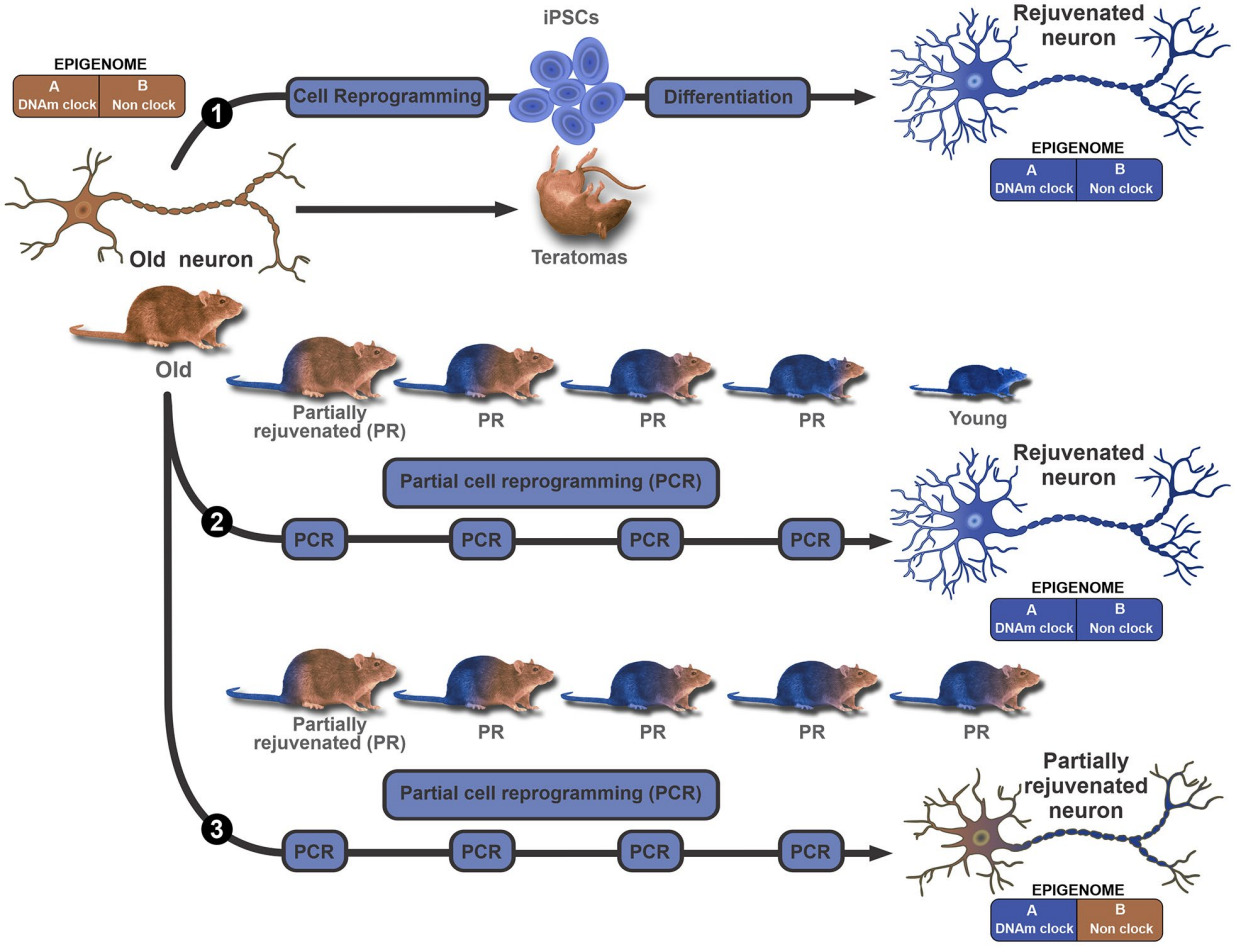
**Modular epigenome model to explain *in vivo* rejuvenation results.** A bimodular epigenome is considered, where Module A represents the DNAm clock component which encompasses all age-dependent DNA methylation epigenetic marks. Module B represents the remainder of the epigenome, including but not limited to cell identity marks. The model does not make further assumptions on the properties of each module. In this context, the existence of, for instance, age-dependent cell identity marks (bivalent marks) is not ruled out. The upper diagram (path 1) proposes that *ex vivo*, conventional reprogramming erases all age and cell identity marks from both modules, and can turn an old (brown color represents old) neuron or any other cell, into an iPSC that can then be differentiated back to a rejuvenated (blue represents young) neuron. *In vivo*, continuous expression of the OSKM genes leads to the genesis of multiple teratomas and the death of the animal. The middle diagram (path 2) illustrates the hypothesis that several cycles of partial reprogramming can progressively rejuvenate cells by erasing all epigenetic marks of age without affecting cell type identity marks. This means that in principle, the strategy could lead to major phenotype rejuvenation *in vivo*. The lower diagram (path 3) illustrates an alternative outcome for partial reprogramming. In this case, partial reprogramming erases age marks from the DNAm clock (module A) but spares age marks of module B. This outcome is compatible with a major resetting of DNAm age but partial rejuvenation of the phenotype.

Although the OSKM genes have been successfully and repeatedly used to rejuvenate somatic cells, the same protocol cannot be used *in vivo* as the dedifferentiation process associated with rejuvenation during cell reprogramming would induce multiple teratomas *in vivo* [45, 46]. This hurdle seems to have been overcome by the development of cyclic partial cell reprogramming, a strategy based on the use of multiple cycles of interrupted reprogramming in which the OSKM genes transcription is turned on briefly and then turned off by means of regulatable promoters (Figure 2-middle and bottom diagrams). In each cycle the process seems to erase some epigenetic marks of age, sparing the epigenetic marks of cell identity [47]. To our knowledge, there are only two documented studies reporting the implementation of cyclic partial cell reprogramming *in vivo*. In one of the studies the treatment, applied to transgenic progeric mice, significantly prolonged their survival and partially rejuvenated some tissues although it did not rejuvenate the mice themselves [48]. In the other study, cyclic partial reprogramming in the hippocampus of middle-aged mice partly reversed the age-dependent reduction in histone H3K9 trimethylation. The treatment elevated the levels of migrating granular cells in the dentate gyrus and also improved mouse performance in the object recognition test [49].

In the studies that achieved cell rejuvenation by means of conventional reprogramming followed by subsequent redifferentiation of resulting iPS cells to the cell type of origin, the phenotype was completely rejuvenated. However, the outcome may not be the same when cyclic partial reprogramming is used. If for instance, it is assumed that the epigenetic pacemaker of aging is a modular mechanism composed of the epigenetic clock (module A) and a non-clock component (module B), then three possibilities can be contemplated a) a default scenario where conventional (full) reprogramming is used. (Figure 2-upper diagram). In this case, all epigenetic marks of age and cell identity are erased leading to a fully rejuvenated phenotype, the iPS cell; b) a partial reprogramming scenario where all epigenetic marks of age are erased from module A and B, but the epigenetic marks of cell identity are fully spared. Conceivably, this could lead to an outcome where the phenotype is fully rejuvenated in every aspect (Figure 2-middle diagram); c) a partial reprogramming scenario where the epigenetic marks of age are erased from module A but not module B; as before, the epigenetic marks of cell identity are fully spared. This could lead to an outcome where the epigenetic clock is set back to a young age, but the phenotype is only partially rejuvenated (Figure 2-bottom diagram). As mentioned above, we are aware of two documented studies reporting the implementation of cyclic partial cell reprogramming *in vivo* [48, 49]. Unfortunately, in neither study epigenetic age was measured which prevented the determination of the extent to which the epigenetic clock was set back.

Despite the promise offered by partial cell reprogramming for safe rejuvenation *in vivo*, it should be pointed out that the OSKM genes are unlikely to have evolved as a physiological mechanism to regulate the epigenetic clock during adult life, rather their most plausible role seems to be the resetting of epigenetic age to zero in the zygote [31].

### Dual effects of Yamanaka genes according to the delivery method

Recent results have revealed that the Yamanaka genes have a dual behavior when expressed continuously *in vivo*, being regenerative when delivered via viral vectors but lethally toxic when expressed in transgenic mice. Thus, a recent report shows that delivery of the OSK genes by intravitreally injecting a regulatable adeno-associated viral vector type 2 (AAV2) expressing the polycistron OSK, can reverse vision deficits in two mouse models [50]. One of them consists of an experimental model of glaucoma in mice. OSK-AAV2 injection into the vitreous body resulted in DOX-responsive OSK gene expression in around 40% of the retinal ganglion cells (RGC) and after 4 weeks of continuous OSK expression, an optometric test revealed a significant recovery of vision which was associated with RGC axon regeneration. The same treatment reversed the typical age-related vison impairment in 12 months old mice. Long-term (12-16 weeks) continuous expression of OSK genes in RGC, induced neither pathological changes nor RGC proliferation. Also, continuous expression (for up to 10-18 months) of OSK in intravenously OSK-AAV2-injected young and old mice was devoid of any adverse side effects. In contrast, DOX-induced expression of OSK in OSK transgenic mice, induced rapid weight loss and death, likely due to severe dysplasia in the digestive system [50]. It is of interest that intravenous OSK-AAV2 vector injection in mice and long-term expression of OSK did not induce weight loss or gastrointestinal abnormalities.

**Figure 3:**
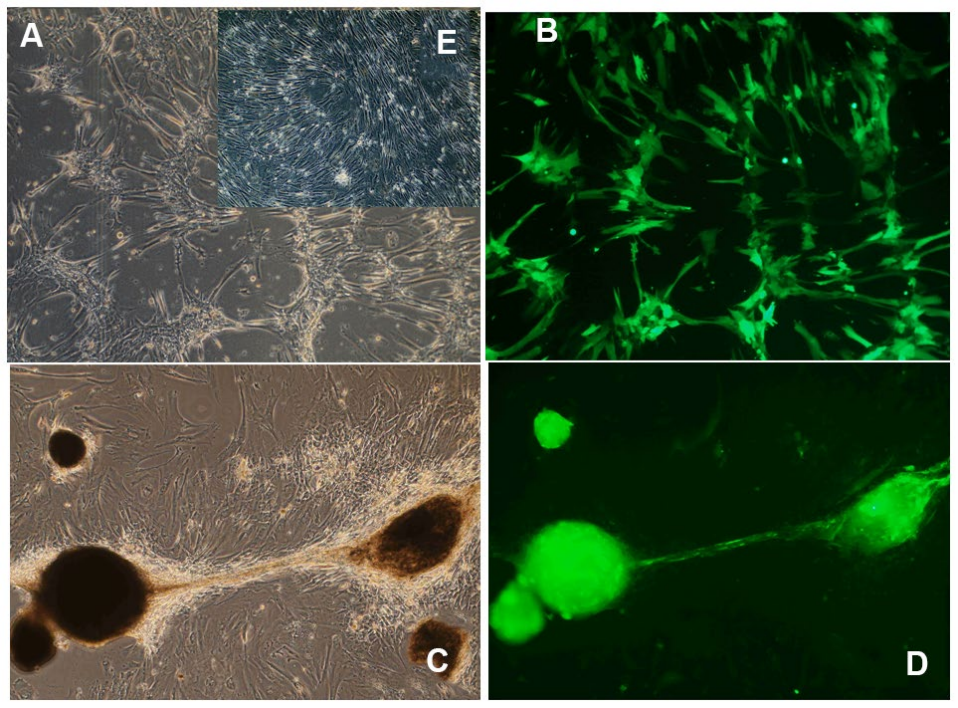
**Morphological changes induced by long-term OSKM gene action in human umbilical cord perivascular cells (HUCPVC).** (**A**) HUCPVC incubated for 7 days with an adenovector expressing a polyscistron harboring OSKM and GFP genes. Phase contrast microscopy; (**B**) The same field observed under fluorescence microscopy. (**C**) HUCPVC incubated for 85 days with the above OSKM-GFP adenovector. Phase contrast microscopy; (**D**) The same field as in C observed under fluorescence microscopy. Inset (**E**) Control intact HUCPVC on Experimental day 7. Obj X 4 in all panels. (Goya et al., unpublished results).

It is well-established that continuous *in vivo* expression of OSKM genes in transgenic mice induces teratomas and other toxic effects that lead to prompt death [38, 44, 47, 48]. Interestingly, in human umbilical cord cells transduced with a Tet-Off regulatable helper-dependent adenovector expressing an OSKM-GFP polycistronic cassette [51], continuous expression of the five transgenes for over 15 weeks, induced neither toxicity nor proliferation although it did induce a significant redistribution of the cells in the cultures (Figure 3). It is also of interest that transient (4 days) transfection of a cocktail of mRNAs expressing Oct4, Sox2, Klf4, c-Myc, Lin28, and NANOG (OSKMLN) induced a rapid reversal of cellular aging in human fibroblasts and endothelial cells, in each case without altering cellular identity. However, longer (15 days) expression of this mRNA cocktail did reprogram the cells [52].

Taken together, these initial results suggest that although the Yamanaka genes remain silent in Tet-On transgenic mice during embryogenesis and early life, transgenesis might in some way sensitize the animals to them when continuously expressed in adult life. In contrast, expression of Yamanaka genes delivered via viral vectors, and expressed continuously, appears to have regenerative activity at least in certain cell types.

Another important implication of the above evidence is that continuous expression of the Yamanaka genes in cells does not necessarily lead to dedifferentiation when the genes are delivered via viral vectors.

### Rejuvenation by non-reprogramming strategies

It has been reported that a one-year combined treatment with human recombinant growth hormone metformin, and dehydroepiandrosterone, of a group of men aged from 51 to 65 years, rejuvenated their epigenetic age by approximately 1.5 years. The treatment, which was designed to rejuvenate the thymus, improved a number of immune parameters [53]. Furthermore, in a recent preliminary study in male rats, results showed that a protein fraction from young plasma can markedly set back the epigenetic age of some tissues in old rats [54]. Specifically, the authors report that repeated intravenous administration of a plasma fraction (termed elixir) from young rats to old counterparts during 5 months (begun at age 20 months until 25 months), sets back the epigenetic age of liver, blood and heart tissue of the treated old rats to nearly that of adult rats (7 months old). The effect of the plasma fraction on the DNAm clock was paralleled by significant functional improvements in a number of hematological, biochemical and functional parameters. The hypothalamus was an exception as the treatment showed a modest although still significant rejuvenation effect on DNAm age. Interestingly, despite the reported improvement of the parameters mentioned above, the old rats were not brought back to a complete young condition. For instance, their body weight, known to increase significantly with age in male rats, was not reduced by the treatment [54]. It seems likely that what this study achieved was a marked reversal of epigenetic age associated with partial rejuvenation of the phenotype. The findings would be compatible with the hypothetical scenario illustrated in the bottom diagram of Figure 2. However, in this particular case a more complete interpretation of results requires a refinement of the model by assuming a trimodular epigenome, where module A encompasses the clock component, module B, represents a non-clock component that is responsive to elixir, and module C represents the portion of the epigenome that is insensitive to the rejuvenating effect of elixir.

## CONCLUSIONS

Gerontology is perhaps the biological discipline that has given rise to the largest number and variety of theories even before the development of modern science. Most theories aimed not only at elucidating the mechanism of aging but also at providing effective interventions to slow aging down. In fact, the field of endocrinology was born from experiments -- aimed at testing a theory of aging -- reported at the end of the XIX century, by Charles E. Brown-Séquard, who injected himself subcutaneously on 10 occasions over a 3-week period, with testicular extracts derived from dogs and guinea pigs in an attempt to counter the effects of aging [55]. In the late 50s the focus of research attention moved to DNA as the likely driver of aging either by expressing a program of aging or by being the target of endogenous and external insults that accumulated damage on the molecule during the lifetime of an organism. Up to this stage, aging was considered as an essentially irreversible process. However, with the discovery of cell reprogramming, early in this century, a view began to emerge that considers aging as a reversible epigenetic process [29–31]. The hypothesis proposing the epigenome as the driver of aging was significantly strengthened by the converging discovery that DNA methylation at specific CpG sites could be used as a highly accurate biomarker of age defined by the Horvath clock [5]. The strong correlation between the dynamics of DNA methylation profiles and the rate of biological aging leads to the idea that the epigenetic clock may in fact be the pacemaker of aging or at least a component of it. And it is at this point where epigenetic rejuvenation comes into play as a strategy to reveal to what extent biological age can be set back by making the clock tick backwards. The few initial results already documented seem to suggest that when the clock is forced to tick backwards *in vivo*, it is only able to drag the phenotype to a partially rejuvenated condition. Nevertheless, it would be premature to draw firm conclusions from the scanty experimental results so far documented. What seems to be clear is that epigenetic rejuvenation by cyclic partial reprogramming or alternative non-reprogramming strategies holds the key to both, understanding the mechanism by which the epigenome drives the aging process and arresting or even reversing organismal aging.

### Ethics approval and consent to participate

All authors agree to publish this article and have accepted to abide by the ethical standards of our Institution.
